# Supplementary material for: Novel Driver Strength Index highlights important cancer genes in TCGA PanCanAtlas patients
Source: PeerJ. 2022 Aug 11;10:e13860. doi: 10.7717/peerj.13860 (PMC9375969; doi:10.7717/peerj.13860)
Supplement: Supplemental Information 2 [file peerj-10-13860-s002.zip › COHORTS/genes plots/age/2021_8_16_14_9_distribution_cohorts_females_40-44.pdf]

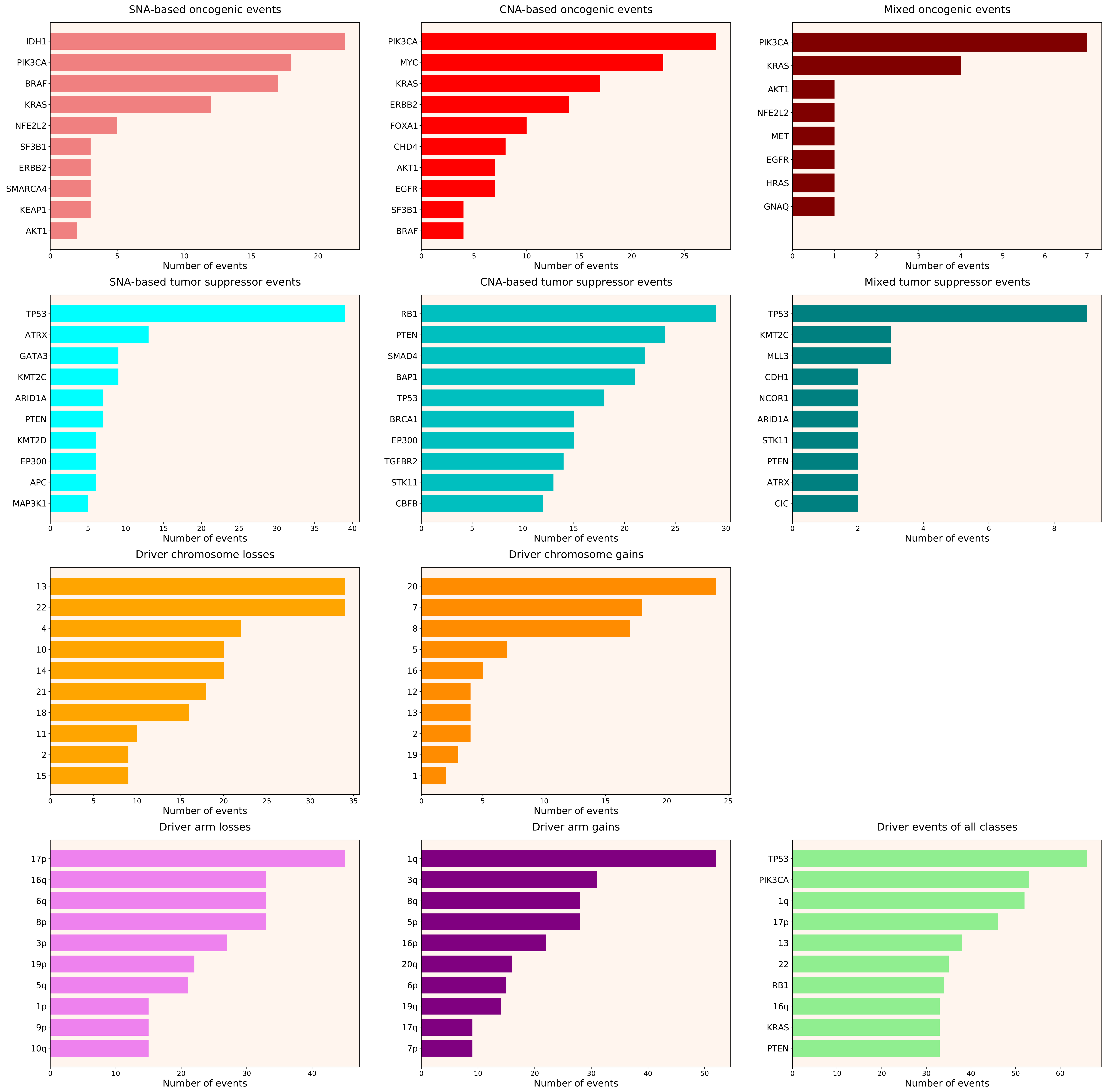

SNA-based oncogenic events
IDH1
PIK3CA
BRAF
KRAS
NFE2L2
SF3B1
ERBB2
SMARCA4
KEAP1
AKT1
Number of events
CNA-based oncogenic events
PIK3CA
MYC
KRAS
ERBB2
FOXA1
CHD4
AKT1
EGFR
SF3B1
BRAF
Number of events
Mixed oncogenic events
PIK3CA
KRAS
AKT1
NFE2L2
MET
EGFR
HRAS
GNAQ
Number of events
SNA-based tumor suppressor events
TP53
ATRX
GATA3
KMT2C
ARID1A
PTEN
KMT2D
EP300
APC
MAP3K1
Number of events
CNA-based tumor suppressor events
RB1
PTEN
SMAD4
BAP1
TP53
BRCA1
EP300
TGFBR2
STK11
CBFB
Number of events
Mixed tumor suppressor events
TP53
KMT2C
MLL3
CDH1
NCOR1
ARID1A
STK11
PTEN
ATRX
CIC
Number of events
Driver chromosome losses
13
22
4
10
14
21
18
11
2
15
Number of events
Driver chromosome gains
20
7
8
5
16
12
13
2
19
1
Number of events
Driver arm losses
17p
16q
6q
8p
3p
19p
5q
1p
9p
10q
Number of events
Driver arm gains
1q
3q
8q
5p
16p
20q
6p
19q
17q
7p
Number of events
Driver events of all classes
TP53
PIK3CA
1q
17p
13
22
RB1
16q
KRAS
PTEN
Number of events
